# Photoactive Titanium Dioxide Films with Embedded Gold Nanoparticles for Quantitative Determination of Mercury Traces in Humic Matter-Containing Freshwaters

**DOI:** 10.3390/nano11020512

**Published:** 2021-02-18

**Authors:** Vivian Stock, Anna Mutschler, Mika Lindén, Kerstin Leopold

**Affiliations:** 1 Department of Analytical and Bioanalytical Chemistry, Ulm University, Albert-Einstein-Allee 11, 89081 Ulm, Germany; vivian.stock@uni-ulm.de; 2 Department of Inorganic Chemistry II, Ulm University, Albert-Einstein-Allee 11, 89081 Ulm, Germany; anna.mutschler@t-online.de

**Keywords:** mesoporous titanium dioxide films, gold nanoparticles, sampling stick, mercury trace analysis, freshwater, humic matter

## Abstract

Mercury detection in humic matter-containing natural waters is often associated with environmental harmful substances for sample preparation. Herein we report an approach based on photoactive titanium dioxide films with embedded gold nanoparticles (AuNP@TiO_2_ dipstick) for chemical-free sample preparation and mercury preconcentration. For this purpose, AuNPs are immobilized onto a silicon wafer and further covered with a thin photoactive titanium dioxide layer. The AuNPs allow the preconcentration of Hg traces via amalgamation, while TiO_2_ acts as a protective layer and, at the same time, as a photocatalyst for UV-C radiation-based sample pretreatment. Humic matter, often present in natural waters, forms stabile complexes with Hg and so hinders its preconcentration prior to detection, causing a minor recovery. This problem is solved here by irradiation during Hg preconcentration onto the photoactive dipstick, resulting in a limit of detection as low as 0.137 ng L^−1^ using atomic fluorescence spectrometry (AFS). A 5 min preconcentration step is sufficient to obtain successful recovery of Hg traces from waters with up to 10 mg L^−1^ DOC. The feasibility of the approach was demonstrated by the determination of Hg traces in Danube river water. The results show no significant differences in comparison with standard cold vapor-atomic fluorescence spectrometry (CV-AFS) measurements of the same sample. Hence, this new AuNP@TiO_2_ dipstick provides a single-step sample preparation and preconcentration approach that combines sustainability with high analytical sensitivity and accuracy.

## 1. Introduction

Mercury is among the most toxic and critical pollutants in our environment. Due to its long lifetime and high mobility, it is distributed globally in the atmosphere and the hydrosphere. Within the latter, Hg bioaccumulates in the food chain, reaching factors of up to 10^6^ from ambient water to predatory fish [1]. Thus, even minor mercury concentrations in the hydrosphere are of interest for early detection and prevention of high mercury levels in human nutrition. Monitoring of Hg in waterbodies is therefore mandatory, and water quality standards are regulated in many countries.

In natural waters, mercury is known to bind strongly to several naturally occurring inorganic and organic ligands, such as dissolved organic carbon (DOC) [2]. Thus, the predominant complex strongly depends on the water type. While in sea water chloro complexes are dominant due to the high salinity (≈19 g L^−1^) and low DOC concentration (<2.5 mg L^−1^) [3,4], freshwaters exhibit comparably lower chloride content (0.2–300 mg L^−1^) [5] and higher DOC concentrations (2–20 mg L^−1^) [6]. Thus, in freshwaters typically the formation of highly stable Hg–DOC complexes is in favor [2]. DOC in freshwater results mainly from the decomposition of biota and is referred to as natural organic matter. Its chemical composition is a complex mixture of large- and small-molecular-weight organic compounds with thousands of different molecules [7,8]. Moreover, the quality and quantity of DOC in natural waters changes spatially and seasonally depending on various factors, such as its soil and vegetation source, biotic and abiotic processing, and storm and drought events [9]. However, humic substances are the major components that are classified into fulvic and humic acids depending on their solubility at different pH values [10].

In Hg trace analysis, the formation of stable complexes with dissolved organic carbon often hinders the quantitative recovery of dissolved mercury, and therefore, sample preparation includes decomposition prior to Hg preconcentration and detection. Standard analytical methods for total mercury determination in waters suggest the addition of strong chemical oxidants prior to cold vapor (CV) generation, preconcentration, and detection [11,12]. Pyhtilä et al. [13] demonstrated that even for most demanding samples, with DOC concentrations of up 130 mg L^−1^, chemical decomposition allows the reliable quantification of Hg traces. However, these approaches require the addition of harmful highly oxidizing substances (e.g., bromine chloride). Moreover, the risk of sample contamination is increased, and therefore, extensive purification procedures are required. Alternatively, reagent-free decomposition of DOC can be achieved using UV radiation, which is an established sample preparation technique in trace element analysis in waters [14]. Irradiation of the sample in the presence of sensitizers like humic acids leads to the formation of highly reactive species, such as singlet oxygen, superoxide radicals, and hydroxyl radicals. These species then decompose large aromatic or aliphatic long-chain molecules into smaller fragments. The decomposition rate can further be enhanced by applying a photoactive titanium dioxide support that either leads to the formation of additional hydroxyl radicals or enables complete and direct oxidation on its surface [15]. In mercury trace analysis, sample pretreatment by UV irradiation has been reported in connection with vapor generation and/or sample matrix decomposition. So-called “photochemical” vapor generation (PVG) has been demonstrated to be applicable in various biological and environmental matrices after the addition of low-molecular-weight organic compounds, like acetic or formic acid [16,17,18]. In natural waters, which originally contain already-small-weight organic acids in the form of humic acids, PVG requires no addition of further chemicals [19,20]. On the other hand, UV-assisted matrix decomposition prior to Hg preconcentration and detection requires the addition of auxiliary reagents, like hydrogen peroxide or other (e.g., [21,22]). Another promising recent trend in Hg trace analysis is the application of nanomaterials (NMs) for efficient and selective preconcentration using solid-phase extraction (SPE) [23]. In this regard, among others, silver nanoparticles (NPs) [24] or nanorods [25], magnetic NPs [26], and thiol-containing nanofibers [27] have been suggested. Most SPE approaches, however, require exact adjustment of adsorption conditions and complex elution procedures. Hence, the necessary set of chemicals and the number of procedure steps then compromise simplicity and often also sensitivity. In order to overcome these drawbacks, the application of gold nanoparticles (AuNPs) for reagent-free preconcentration of dissolved mercury species and subsequent thermal desorption has been suggested by our group [28,29]. The adsorption of mercury species can then be explained by a three-step mechanism induced by the catalytic activity of the nanogold that promotes (1) stripping of any alkyl groups (in the case of alkylmercury, e.g., methylmercury), (2) reduction of mercuric ions, and finally, (3) amalgamation of elemental mercury (for details, please refer to [30]). Hence, AuNP-based SPE materials provide a fully reagent-free procedure for Hg trace analysis in waters and are therefore predestined for combination with reagent-free DOC decomposition. To the best of our knowledge, there are no reports on decomposition approaches that take advantage of titanium dioxide photocatalytic enhancement in the field of mercury trace analysis. Hence, in this work we aim to combine the benefits of the TiO_2_ thin film, providing photoactive decomposition of dissolved humic matter with the highly selective and efficient preconcentration of Hg by amalgamation onto AuNPs. Moreover, for the purpose of convenient and easy handling, preconcentration is performed via solid-phase extraction onto a striplike sampling device, a “sampling stick.” For long-term monitoring of environmental mercury levels, such sampling approach is well-known as diffusive gradient in thin film (DGT) probes. However, DGT probes are deployed for several days up to weeks in the field and allow only for an estimation of Hg level in the waterbody (see, e.g., [31]). Measuring the actual Hg concentration after short-time accumulation (i.e., deployment of a sampling stick for only a few minutes) is rarely described in the literature. First approaches using immobilized AuNPs onto different support materials, as reported by the authors, showed very promising analytical performance. However, at the same time, poor mechanical stability and limited lifetime were observed [32,33]. Therefore, the development of a more sophisticated structure of the active film covered by a protective layer was necessary. The latter has already been shown in a recent work of the authors, where we reported about mesoporous silica–gold films for efficient chemical-free Hg trace analysis [34]. Parameters found optimum for mercury accumulation in terms of film thickness and mesopore diameter in that study are used as design targets for the current study.

## 2. Materials and Methods

### 2.1. Chemicals

Ultrapure water (UPW) with a resistivity of 18.2 MΩ cm was used for the preparation of solutions and dilution analytical standards using mercury standard solution (traceable to SRM from NIST, Hg(NO_3_)_2_ in HNO_3_, 2 mol·L^−1^, Merck, Darmstadt, Germany). For atomic fluorescence spectrometry (AFS) measurements, argon gas (99.996%, MTI IndustrieGase AG, Ulm, Germany) was used as a carrier gas. Reference measurements according to the EPA method 1631 required potassium bromide (VWR International GmbH, Darmstadt, Germany), potassium bromate (VWR International GmbH), hydroxylammonium chloride (VWR International GmbH), hydrochloric acid, 37% (p.a. EMSURE^®^, Merck, Darmstadt, Germany), and tin(II) chloride (≤0.000001% Hg, p.a. EMSURE^®^, Merck, Darmstadt, Germany). DOC-containing model solutions were prepared from dilution of Suwannee River Humic Acid Standard III (3S101H, International Humic Substance Society) and Suwannee River Fulvic Acid Standard III (3S101F, International Humic Substance Society). For synthesis of the thin films, ethanol (Merck, absolute for analysis), Pluronic^®^ F-127 (Sigma-Aldrich, Darmstadt, Germany), and titanium tetrachloride (Merck) were applied. Moreover, demineralized water and synthetic air gas mixture (20.5 Vol-% oxygen, rest nitrogen) were used.

### 2.2. Preparation of Gold-Nanoparticle-Covered Silicon Wafers

As a substrate for the films, Si wafers (Silicon Materials, P/Bor<100>, Kaufering, Germany) were used. First, 4 × 0.5 cm^2^ pieces were cut and cleaned by immersion in acetone and ultrasonic treatment. Then, the substrates were cleaned in a warm acetone bath, followed by an isopropanol and ultrapure water bath, and finally dried under an ultrapure N_2_ flow. These steps were performed in a clean room, where vacuum evaporation of the gold layer was carried out subsequently. As a result, a uniform gold layer with a thickness of about 6 nm was obtained. This thickness was found to be optimum with regard to nanoparticle formation, coverage of protective film, and high gold load, as tested previously [34]. The formation of about 30 nm gold nanoparticles on the silicon substrates was induced by heating the wafers in air to 270 °C on a hotplate for 2 h.

### 2.3. Preparation of the Mesoporous Titanium Dioxide Top Layer

#### 2.3.1. Sol Preparation

Regarding the instructions for the preparation of the sol, the followed dip-coating process and the thermal treatment are modified from Crepaldi et al. [35]. In brief, 1.06 g Pluronic^®^ F-127, used as the structure-directing agent, was dissolved in 37 mL ethanol (abs.) in a 100 mL round-bottomed flask. To this solution, 1.75 mL titanium tetrachloride and 2.84 mL demineralized water were added under stirring (500 rpm). The mixture was aged for another 24 h at room temperature under stirring with 500 rpm before use.

#### 2.3.2. Dip-Coating Process

For the dip-coating process of the silicon wafers carrying an array of gold nanoparticles, the humidity regulation system coupled to the dip coater was first adjusted to a relative humidity of about 30%. Afterwards, the dip chamber was filled with the sol, and the dip coater was purged again with the set relative humidity. Next, the substrates were dipped into the sol and drawn continuously from the sol with a drawing speed of 0.5 mm·s^−1^. The air flow was stopped during the dipping process, and after the substrates were removed from the sol, the air flow was waited for 1 min and then restarted with a relative humidity of about 50%. The substrates were allowed to dry for a few minutes at the set relative humidity in the dip coater and were then placed in a chamber, which was purged with synthetic air with a relative humidity of about 50%, and aged there for 2 days.

#### 2.3.3. Thermal Treatment

After aging, the films were treated at different temperatures for crystallization. First, they were heated to 60 °C with a heating ramp of 1 °C·min^−1^ and treated for 24 h. Then the same heating ramp was used to heat them to 100 °C, and the temperature was held for 24 h. Afterwards, a temperature of 130 °C was set with a heating ramp of also 1 °C·min^−1^ and held for 24 h. Finally, the films were calcined to open the pores and remove the organic components. Therefore, again a heating ramp of 1 °C·min^−1^ was used to heat the films from 130 to 400 °C. The temperature was kept for 4 h.

### 2.4. Film Characterization

Ready-made AuNP@TiO_2_ films were characterized using scanning electron microscopy (SEM; Helios NanoLab 600, ThermoFisher Scientific, Germany, operated at 5 kV). In addition to top-view images, cross-sectional images were prepared using the integrated focused ion beam (FIB) for milling. For this purpose, first, a layer of platinum was sputtered onto the area of interest in order to avoid electrostatic charging of the sample. High-resolution images for the investigation of the mesoporous structure of titania films were obtained by transmission electron microscopy (TEM; Jeol 1400, JEOL (Germany) GmbH, Freising, Germany, operated at 120 kV) using a removable aluminum foil as a substrate. Here, embedding in an epoxy resin was required before the substrate was removed, followed by an additional epoxy resin treatment and cutting into thin slices. The identification of the TiO_2_ structure was achieved using X-ray diffraction measurements (Panalytical X’Pert Pro system equipped with an X’Celerator detector, operated in reflection mode, Malvern, UK).

### 2.5. Investigation of the Stability of Gold in the Films

The gold stability of the dipstick was verified by total reflection X-ray fluorescence (TXRF; S2 Picofox, Bruker AXS GmbH, Berlin, Germany) measurements of sample solution after dipstick exposure for 5 min at 230 rpm. For this purpose, an aliquot of 990 µL of the sample was taken, and 10 µL of a 100 µg L^−1^ vanadium standard was added as internal standard. The solution was thoroughly mixed, and then 10 µL was applied onto a silicone-coated quartz glass carrier, which was subsequently placed onto a heating plate (70 °C) for complete evaporation of the solvent. After drying of the sample, TXRF measurement was performed using a tube voltage of 50 kV, current rating of 600 µA, and lifetime of 1000 s. The lower limit of detection of Au was 0.1 µg L^−1^.

The gold load of the TiO_2_ films was determined after gold extraction in 5 mL aqua regia. To ensure that gold was quantitatively extracted from the dipstick, gold extraction was repeated once. An amount of 75 µL of each extractant solution was then diluted in 4.9 mL UPW. Then 25 µL of a 10 mg L^−1^ vanadium standard was added as internal standard, and the solutions were mixed thoroughly. Subsequently, an aliquot of the diluted extraction solution was applied onto a silicone-coated quartz glass carrier and measured by TXRF (S2 Picofox, Bruker AXS GmbH, Berlin, Germany) as described above.

### 2.6. Mercury Accumulation and Investigation of Analytical Performance for Hg Quantification

Mercury accumulation experiments were performed in glass cylinders equipped with a specially designed cap in which the sampling stick can be fixed. These containers were filled with either 6 mL or 8 mL of a Hg^2+^-containing aqueous solution, and accumulation was performed at room temperature on an orbital shaker at 230 rpm for 5 min. These parameters were tested in our previous study and found to be optimum [34]. Hg concentrations ranged from 5 to 25 ng L^−1^. For measurements in humic substances containing model solutions with dissolved organic carbon (DOC), concentrations of 0, 5, 10, and 15 mg L^−1^ were prepared by adequate dilution of fulvic acid stock solution. This was prepared by dissolving 18.76 mg fulvic acid standard (Suwannee River III) provided by the International Humic Substance Society (IHSS, Denver, CO, USA) in 100 mL UPW. The suspension was then passed through a 0.45 µm pore-size polyether sulfone syringe filter and acidified with 0.5% (*v/v*) HCl. Fulvic acid (FA) stock standard was kept in the dark at 4–7 °C for a maximum of 2 weeks.

Experiments with UV-C irradiation during the 5 min Hg accumulation were performed in a UV-transparent quartz glass container using a UV lamp (10 W, Peschl UV-Consulting, Mainz, Germany) with two emission maxima at λ_1_ = 189 nm and λ_2_ = 254 nm.

After accumulation, sampling sticks were removed from the caps, rinsed with UPW, and placed in a heatable collector tube coupled online to an atomic fluorescence spectrometer (AFS; Mercur, Analytik Jena AG, Jena, Germany). For thermal mercury release from the sticks, the collector tube is heated, and an argon carrier gas transports Hg vapor to the AFS measurement cell. The timeline of this online procedure and other parameters were set as previously reported in detail by Mutschler et al. [34]. Briefly, AFS measurements were performed at a wavelength of 253.7 nm and a detection voltage of 391 V. External calibration of the AFS was performed using the cold vapor technique (CV) in order to determine Hg masses released from the dipsticks. Elemental Hg^0^ vapor was generated online using a reduction solution of 1.25% (*v/v*) HCl containing 0.65% (*w/v*) SnCl_2_ and a carrier solution of 0.5% (*v/v*) HCl. Hg^2+^ standards were prepared freshly by adequate dilution of a stock standard. The sample volume used for calibration was 2.3 mL. The external calibration function is given as y = 0.000813 L·ng^−1^ x + 0.000183 (*R*^2^ = 0.9995). The resulting limit of detection for CV-AFS is 0.255 ng Hg L^−1^.

### 2.7. Investigation of Real River Water Sample

Proof of principle was performed using a real freshwater sample. The sample originates from the river Danube (48° 23′ 09.5′′ N 9° 59′ 01.0′′ E) and was collected in August 2020 in Ulm, Germany. For sampling, precleaned glass containers were rinsed three times with the sample before they were filled without headspace. The river water sample was immediately transported to the laboratory, where it was filtered using a 0.45 µm pore-size filter and acidified with 0.5% (*v/v*) HCl. The sample was stored in the dark at 4–7 °C until usage. Natural DOC concentration was determined at the Department of Civil, Geo, and Environmental Engineering of the Technical University of Munich according to the standard method EN 1484, DEV H3 [36], using a TOC analyzer (VarioTOC, Elementar, Germany). Validation of the found mercury concentration in the sample was achieved by the application of the standard CV-AFS reference method for total dissolved mercury traces in waters according to the U.S. EPA method 1631 [12].

## 3. Results and Discussion

### 3.1. Motivation for the Design of New Nanogold Mesoporous Titanium Dioxide Films

**Figure 1:**
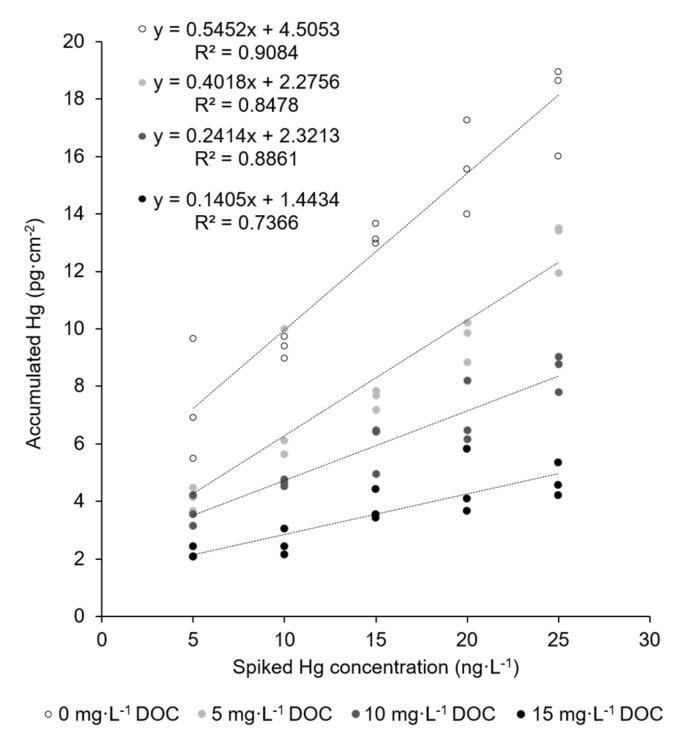
Accumulation of mercury traces onto the AuNP@SiO_2_ dipstick in the absence (empty circles) and presence (filled circles) of different concentrations of DOC. Sample volume: 8 mL; accumulation time: 5 min; shaking speed: 230 rpm; active dipstick area: 1.53 cm^2^.

Recently, the authors reported on the straightforward trace-level monitoring of mercury in water using mesoporous silica–gold films (AuNP@SiO_2_) [34]. Here, a film of gold nanoparticles with a mean particle diameter of about 30 nm was first produced on a silicon wafer substrate through temperature-driven dewetting. This gold nanoparticle array was then covered by a 100 nm thick film of mesostructured silica (space group *Im-3m*) using Pluronic^®^ F-127 as the structure-directing agent through dip coating. The surfactant was removed by calcination, and the resulting mesoporous film had a pore diameter of about 6–7 nm as determined by transmission electron microscopy (TEM) imaging. The mercury trace-level determination using these films for mercury accumulation was validated by the measurement of the certified reference material ORMS-5, a river water. However, new measurements of a series of real river water samples revealed that quantitative Hg recovery is not always possible. In contrast to the studied reference material, real river waters were not stabilized using highly oxidative BrCl solutions. Therefore, a plausible explanation could be the presence of dissolved organic carbon (DOC) in some river waters coming from natural humic matter. DOC is decomposed by BrCl treatment, while it is stabile using only HCl acidification. Thus, in a series of experiments, the influence of naturally occurring organic compounds on the mercury extraction efficiency onto the AuNP@SiO_2_ dipstick was investigated. For this purpose, a fulvic acid standard (Suwannee River III) extracted from river water and derived from the International Humic Substance Society was used to prepare model solutions with known DOC concentrations. As fulvic acids belong to the hydrophobic acids and have a high proportion of reduced sulphur sites, which are the main groups to interact with mercury in natural freshwater, the fulvic acid standard should be a good representative for DOC in this case. The results are shown in Figure 1 and confirm that increasing DOC concentrations lead to a decreasing amount of accumulated Hg on the AuNP@SiO_2_ dipsticks.

Even at the lowest tested DOC concentration of 5 mg L^−1^, recovery of mercury decreases to 74%. In natural waters, however, DOC concentrations vary in a broad range and can be as high as 20 mg L^−1^ in humic-rich freshwaters [4,6,37]. Obviously, digestive sample pretreatment (i.e., addition of BrCl prior to accumulation) could resolve this problem and make the application of the AuNP@SiO_2_ dipstick possible. However, we aim to provide a reagent-free method that provides sustainable and simple Hg trace monitoring that can also be performed on site. Handling of highly toxic and environment-impairing BrCl solution was therefore to be avoided. Reagent-free decomposition of dissolved organic carbon in natural waters has been shown using UV irradiation, which can be further enhanced by photoactive titanium dioxide support [15]. Therefore, a new dipstick type was designed, prepared, and characterized in which the mesoporous silica film was replaced by a titanium dioxide film.

### 3.2. Synthesis and Characterization of Titanium Dioxide-Embedded Gold Nanoparticle Substrate

**Figure 2:**
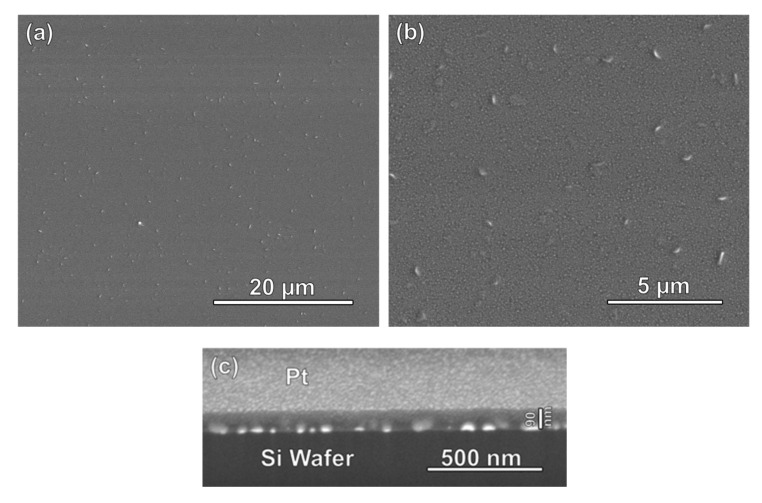
(**a,b**) SEM images (top view) of a gold nanoparticle array on a silicon wafer homogeneously covered by a film of mesoporous titania. (**c**) Focused ion beam (FIB)-SEM cross-sectional image of the same film. The gold nanoparticles are seen as bright spots, and these are fully covered by the mesoporous titania film, which has a thickness of about 90 nm. For FIB milling, the titania film is covered with a conductive layer of Pt, which gives a brighter contrast in comparison with the Si substrate.

Scanning electron microscopy (SEM) images of the prepared AuNP@TiO_2_ films on a silica substrate are shown in Figure 2a,b. Despite the uneven surface of the gold-nanoparticle-covered silicon wafers, the mesoporous titania film is homogeneous, but the higher-resolution SEM image reveals the presence of evenly distributed local thickness maxima with a mutual distance of some 2 μm and a length of some 100 nm and a width of about 20 nm. These maxima probably originate from a combination of the uneven support due to the presence of the gold nanoparticles and tensions in the film induced during crystallization of the originally amorphous TiO_2_ into anatase upon thermal treatment (see later). A cross-sectional FIB-SEM image of the film is shown in Figure 2c. Here, the gold nanoparticles are clearly seen as bright spots, and these are homogeneously covered by the titania film, which has a thickness of about 90 nm.

**Figure 3:**
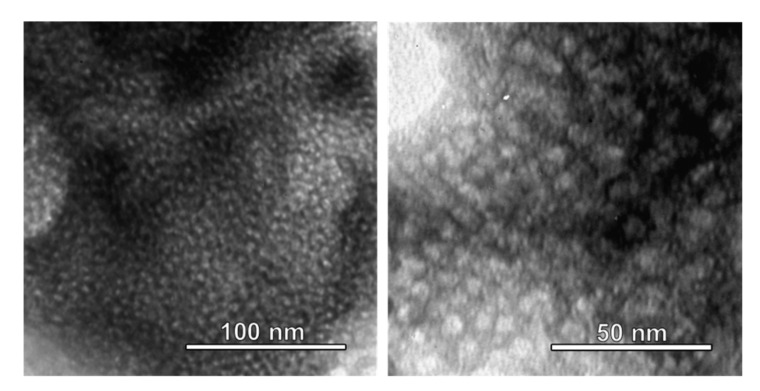
TEM images of mesoporous titania films. Mesopores with a mean diameter of about 6–7 nm are seen to be homogeneously distributed throughout the film.

TEM images taken of the mesoporous titania film are shown in Figure 3. Quasi-spherical mesopores with a mean diameter of about 6–7 nm are homogeneously distributed throughout the film. No long-range order can be observed, which is in full agreement with low-angle X-ray diffraction results that do not show any signs of periodicity on the length scale corresponding to the mesopores (results not shown).

**Figure 4:**
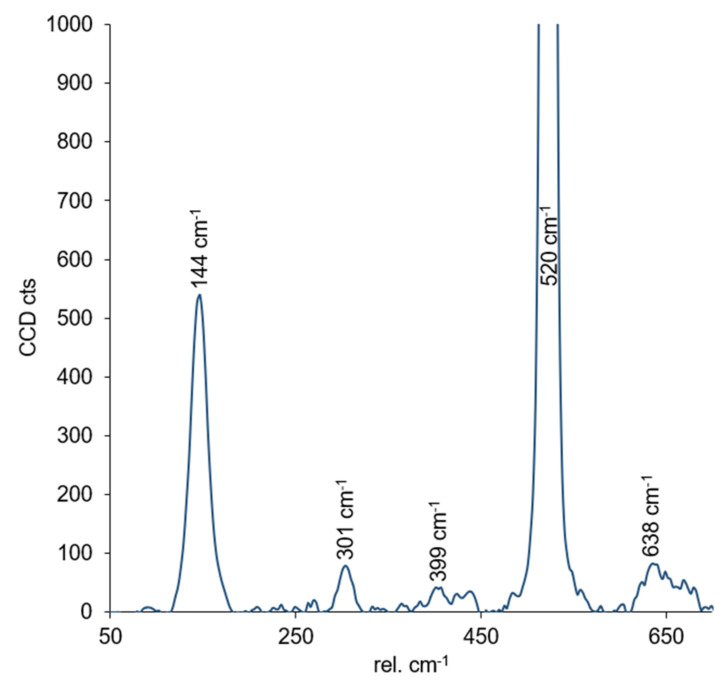
Raman spectrum measured for the titania films deposited on a silicon wafer. See text for details.

Raman spectroscopy measurements were carried out in order to determine the crystallographic order of the titania. The Raman frequencies for anatase are 144 cm^−1^ (E_g_)*, 197 cm^−1^ (E_g_), 399 cm^−1^ (B_1g_)*, 513 cm^−1^ (A_1g_), 519 cm^−1^ (B_1g_)*, and 639 cm^−1^ (E_g_)* and 143 cm^−1^ (B_1g_)*, 447 cm^−1^ (E_g_)*, 612 cm^−1^ (A_1g_)*, and 826 cm^−1^ (B_1g_) for the rutile phase [38]. The measured Raman spectrum for the titania films on a silicon wafer support is shown in Figure 4. The bands located at 301 cm^−1^ and 520 cm^−1^ can be assigned to the silicon wafer [39]. As can be seen, all other Raman bands can be assigned to the anatase phase.

Thus, the mesoporous anatase titania–gold films do exhibit virtually identical structural characteristics as the mesoporous silica–gold films discussed in [34], as both the mesopore diameters and the film thicknesses are comparable. Furthermore, gold nanoparticle size estimates based on the full width at half maximum (FWHM) of the 111 reflection at 38.1° 2θ yielded a mean crystallite size of about 30 nm, in perfect agreement with that observed for the gold–silica films.

### 3.3. Comparison of Performance of Silica and Titanium Dioxide-Based Nanogold Films

First, Mutschler et al. [34] demonstrated that a crack-free SiO_2_ film works as a protective layer keeping AuNP from being washed off the dipstick during Hg accumulation and rinsing procedure. These findings were confirmed for the TiO_2_ film too. This is the result of an investigation of the accumulation solutions by total reflection X-ray fluorescence (TXRF) spectrometry, which gave no gold signals. Moreover, gold extraction in aqua regia from a dipstick that was used for 16 measurement cycles confirms these findings. Consistent with the previous experiment, 11.02 ± 0.48 µg cm^−2^ was extracted from the sampling stick, which agrees with the mass that was deposited initially (11.11 ± 0.22 µg cm^−2^).

**Figure 5:**
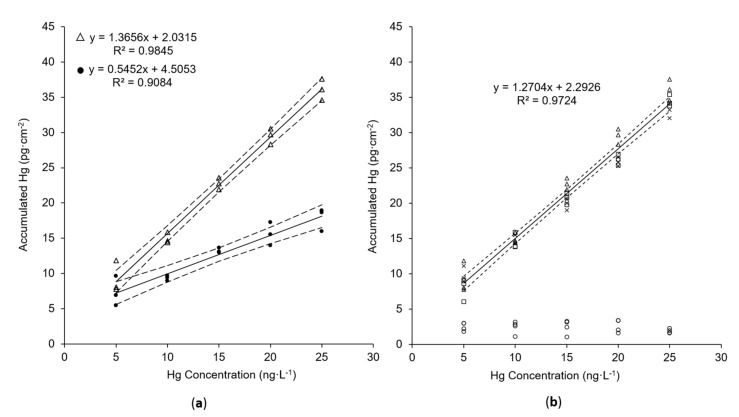
Comparison of Hg accumulation using (**a**) AuNP@TiO_2_ sampling stick (Δ) or AuNP@SiO_2_ sampling stick (•) and (**b**) different batches of AuNP@TiO_2_ sampling stick (Δ, □, x) or blank TiO_2_ sampling stick (○). V= 8 mL (Δ, •) or 6 mL (□, x, ○); t_acc_= 5 min; shaking speed: 230 rpm. Confidence intervals (dashed lines) were calculated with *p* = 95% and *n* = 15 in (**a**) and *n* = 45 in (**b**).

Hg accumulation onto the new AuNP@TiO_2_ dipsticks is compared with the previously presented mesoporous silica–gold film in concentration-dependent measurements. For this purpose, both dipstick types—in the following referred to as AuNP@SiO_2_ and AuNP@TiO_2_ sampling sticks—were immersed in aqueous solution containing known concentrations of dissolved HgCl_2_ in the ng L^−1^ level. All experimental parameters that are known to significantly influence the accumulated amount of mercury were kept identical, like active gold area (A_active_), accumulation time (t_acc_), temperature, and shaking rate. For quantification of the accumulated amount of mercury, Hg^0^ is thermally desorbed from the sticks and measured by AFS as described in the experimental section. As can be seen in Figure 5a, the slope of the linear regression, and thus the sensitivity of the new AuNP@TiO_2_ sampling stick, is 2.5 times higher in comparison with the AuNP@SiO_2_ sampling stick. Moreover, a significantly lower standard deviation of the calibration procedure of 6.35% instead of 16.08% (AuNP@SiO_2_) indicates higher precision of the new sampling stick. Nevertheless, a constantly low blank signal was obtained from a blank TiO_2_ sampling stick (i.e., one without nanogold) (see Figure 5b, circles). This indicates that accumulation is still quasi-exclusively driven by AuNPs. Since the gold load of 11.11 ± 0.22 µg cm^−2^ found for the new AuNP@TiO_2_ sampling stick is comparable to 10.26 ± 0.17 µg cm^−2^ [34] found for the AuNP@SiO_2_ sticks, the observed enhanced performance of the AuNP@TiO_2_ sticks must be based on a better accessibility of the AuNPs. The reasons for this could be structural changes occurring preferentially in the silica films related to the dissolution–reprecipitation process leading to a lower porosity over time, while such processes are not expected to be dominant for the titania films as the solubility of titania is clearly lower than that of amorphous silica.

Furthermore, the reproducibility of Hg accumulation using different synthesis batches of the new sampling sticks was tested. No significant differences in the obtained accumulated Hg masses were obtained (see Figure 5b). The standard deviations calculated from the three batches and three replicates vary between 15.2% (for 5 ng L^−1^) and 4.7% (for 25 ng L^−1^), confirming high reproducibility.

**Figure 6:**
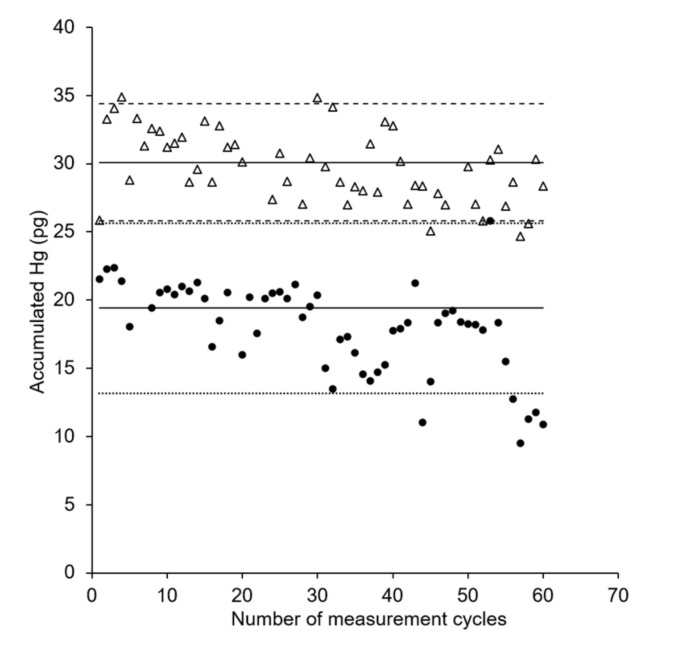
Comparison of lifetime of AuNP@SiO_2_ (•) and AuNP@TiO_2_ (Δ) sampling stick. V = 6 mL; c(Hg): 15 ng Hg L^−1^; t_acc_ = 5 min; shaking speed: 230 rpm. Solid lines represent the expected amount of accumulated mercury as derived from the first calibration of a sampling stick. Dashed lines represent variation ranges as derived from the residual standard deviation (SD) of the calibration with a coverage factor *k* = 2.

In order to study the lifetime of the AuNP@TiO_2_ sampling stick in comparison with the AuNP@SiO_2_ sticks, repeated measurements at a constant mercury concentration using the same sampling stick were performed for each type over multiple days. The AuNP@SiO_2_ sampling stick gives stable responses for approximately 40 measurement cycles before a decrease in efficiency occurs (see Figure 6, circles). This is in agreement with our previous findings for this dipstick type [34]. In contrast, the new AuNP@TiO_2_ sampling stick shows a lifetime of at least 60 measurement cycles, as constant mercury accumulation for 60 successive measurements was observed (see Figure 6, triangles).

In conclusion, the new AuNP@TiO_2_ sampling stick is superior in its analytical performance to the previously described silica-based stick. Hence, the most important question is whether the reagent-free quantitative determination of mercury traces in DOC-containing solutions is possible with the new sampling stick.

### 3.4. Mercury Accumulation from Dissolved Organic Carbon Model Solutions

In a further series of experiments, the influence of naturally occurring DOC on the mercury extraction efficiency onto new the AuNP@TiO_2_ dipstick was investigated. Analogous to the experiments described in Section 3.1, Suwannee River III extract was used to prepare model solutions with known DOC and Hg concentrations. As can be seen in Table 1, mercury recovery in the presence of DOC decreases significantly without irradiation, while quantitative recovery is achieved under UV-C irradiation up to a concentration of 5 mg/L^−1^ DOC. For Hg solutions containing 10 mg/L^−1^ DOC, the recovery was enhanced to approximately 83% using UV-C-assisted digestion, which is still acceptable for ultra-trace analysis.

In contrast, Hg accumulation onto the previously described AuNP@SiO_2_ dipsticks could not be improved by UV-C irradiation in this significant way (see Supplementary Materials Figure S2). Hence, the photoactive TiO_2_ film enhances the analytical performance of the new dipsticks significantly with regard to Hg determination in freshwaters. Therefore, the applicability of the sampling sticks can be broadened to a wide range of natural freshwater samples, such as river waters, providing a chemical-free procedure.

### 3.5. Proof of Principle and Analytical Figures of Merit

For proof of principle, the above-described procedure was applied to a nonspiked natural Danube river water sample. First, a thorough characterization of the water matrix was performed. The results are given in Table 2.

The conductivity, temperature, and pH of the sample are in good agreement with a general characterization of the upper Danube river water as stated by the International Commission for the Protection of the Danube River [40]. The found DOC concentration is slightly lower than one would expect. This might be due to the specific sampling spot that was located just after the confluence of the river Iller. Similarly, an investigation of the river water using the standard reference EPA method 1631 (including oxidative digestion) [12] for the determination of mercury reveals an extra-low Hg concentration of 0.41 ng L^−1^ range. Hence, the found DOC concentration of approximately 2 mg L^−1^ presents an even higher proportional excess in comparison with the tested standard solutions (see Table 1). Anyway, all parameters of the accumulation process were kept as given above, and the Hg concentration was determined using the new AuNP@TiO_2_ dipstick in combination with UV-C irradiation. The mercury concentration found by this procedure was 0.49 ± 0.07 ng Hg L^−1^. Thus, no significant difference for the value found by the reference method was observed, and the applicability of the new approach to Hg ultra-trace analysis in river water is successfully proven.

Finally, analytical figures of merit were determined for the new approach and are summarized in Table 3 in comparison with those previously reported for the AuNP@SiO_2_ dipstick.

The change of the protective film material from SiO_2_ to TiO_2_ of the nanogold-based dipstick clearly enhances its analytical performance. While precision and batch-to-batch reproducibility are maintained, the new composite material provides much better sensitivity, which is reflected in an almost six times lower detection limit. With a limit of quantification as low as 0.274 ng L^−1^ and a tolerable DOC content of up to 10 mg L^−1^, reagent-free ultra-trace analysis in pristine natural freshwaters is possible with this approach.

### 3.6. Comparison of Performance with Other NM-Based Solid-Phase Extraction Approaches

The data obtained are comparable to or even better than the values reported recently for other NM-based SPE approaches applied to natural waters for total mercury preconcentration, as can be seen in Table 4. Moreover, it is noteworthy that the suggested sampling stick procedure here is the only method combining sample pretreatment and preconcentration in a single and reagent-free step providing sustainability, easy handling, and at the same time, high analytical performance.

## 4. Conclusions

Herein we report for the first time a preconcentration procedure for sustainable, reagent-free, and easy-handling mercury ultra-trace analysis in natural freshwaters. A straightforward and highly reproducible preparation of nanocomposite material in the form of a sampling stick consisting of AuNPs embedded in a porous anatase titania thin film deposited onto a silicon substrate is described. A thorough characterization of the AuNP@TiO_2_ sampling stick reveals a crack-free effective TiO_2_ protection layer for the stabilization of the AuNPs during sample exposure. The anatase structure with an average pore size of about 6–7 nm shows no long-range order. A good batch-to-batch reproducibility of the nanocomposite material was observed. In comparison with the previously reported mesoporous silica–gold film [34], the new sampling stick provides much better analytical performance, such as higher sensitivity, higher lifetime, and precision. Accordingly, a detection limit as low as 0.137 ng L^−1^ was reached, and standard deviations are in the range of 3%. The sustainability of the suggested approach is given by the reusability of the sampling stick for more than 60 measurement cycles and the fact that the complete procedure of preconcentration and detection is reagent-free. Most importantly, UV-assisted sample pretreatment is significantly enhanced by the photoactive TiO_2_ thin film, which allows, even in DOC-containing freshwaters, Hg ultra-trace analysis without the addition of any chemicals. This was evidenced by the Hg trace determination in model solutions of dissolved organic carbon with up to 10 mg L^−1^ DOC. Moreover, an accurate determination of Hg ultra traces in a nonspiked real river water from Danube was achieved, as proven by the successful comparison of the results obtained from a standard measurement method. In conclusion, this new approach, on the basis of the AuNP@TiO_2_ sampling stick, provides sample preparation and preconcentration in one reagent-free step, providing a method for simple, accurate, and sustainable Hg trace monitoring in freshwaters.

## Figures and Tables

**Table 1:** Mercury recovery in DOC-containing solutions using the new AuNP@TiO_2_ dipstick with and without UV-C irradiation. Recovery rates are derived from the evaluation of the recovery function in a Hg concentration range from 5 to 25 ng L^−1^; uncertainties are calculated from the confidence intervals of the recovery functions with *p* = 95% and *n* ≥ 12. For the full dataset, please see Supplementary Materials Figure S1.

DOC Concentration/ mg L^−1^	No UV Irradiation	UV Irradiation
	Recovery Rate (%)	Recovery Rate (%)
5	68.6 ± 11.0	106.0 ± 11.8
10	6.0 ± 5.9	82.6 ± 6.6
15	12.4 ± 2.7	63.4 ± 8.1

**Table 2:** Characteristics of a Danube river water sample as determined in situ if not stated otherwise.

pH	8.27
Temperature (°C)	22.8
Conductivity (µS cm^−1^)	558
Total dissolved solids (TDS) (mg L^−1^)	394
Salinity (ng L^−1^)	0.28
DOC ^a)^ (mg L^−1^)	1.6 ± 0.05
Hg ^b)^ (ng L^−1^)	0.41 ± 0.03

^a)^ As determined by the standard method DIN EN 1484:2019-04 in the laboratory [36]; ^b)^ as determined by the standard reference method U.S. EPA 1631 [10] in the laboratory.

**Table 3:** Analytical figures of merit for reagent-free Hg determination using the new AuNP@TiO_2_ dipstick and UV-C irradiation in comparison with those previously reported for the AuNP@SiO_2_ dipstick. Experimental parameters: V = 8 mL, t_acc_ = 5 min, A_active_ = 1.53 cm^2^, *n* = 3, *p* = 95%.

Parameters	New AuNP@TiO_2_ Dipstick (This Work)	Previously Reported AuNP@SiO_2_ Dipstick [34]
Linear working range ^a)^ (ng L^−1^)	0.1–100	1–100
Regression coefficient *R*^2^	0.9966	0.9816
Precision given at different Hg levels as RSD (%)		
c(Hg) = 0.6 ng L^−1^	3.22	-
c(Hg) = 1.2 ng L^−1^	3.20	2.39
c(Hg) = 15 ng L^−1^	3.54	2.20
Accuracy as recovery in Danube river water ^b)^ (%)	118 ± 19	-
Accuracy as recovery in ORMS-5 (%)	-	92.0 ± 9.8
Sensitivity (slope of calibration function) (L ng^−1^)	0.0015	0.0003
Limit of detection ^c)^ (ng L^−1^)	0.137	0.753
Limit of quantification ^c)^ (ng L^−1^)	0.274	1.51
Recyclability of sampling stick (cycles)	≥ 60	≥ 30

Notes: ^a)^ Higher concentration was not tested in order not to contaminate the analytical setup for trace analysis; ^b)^ as derived from the reference measurement using the standard reference EPA method 1631 [12]; ^c)^ calculated on the basis of the obtained calibration function according to Hubaux and Vos [41].

**Table 4:** Comparison of performance with other solid-phase extraction approaches using nanomaterials (NMs). Abbreviations: IL-GO, graphene oxide functionalized with an ionic liquid; Fe_3_O_4_@SiO_2_SiDTC, iron oxide nanoparticles coated with silica shells functionalized with dithiocarbamate groups; APDC, ammonium pyrrolidine dithiocarbamate).

	AuNP@TiO_2_ [This Work]	IL–GO Hybrid NM [42]	APDC/Graphene Nanosheets [43]	Fe_3_O_4_@SiO_2_SiDTC [44]
Precision (%)	≤3.5	3.9	<4.5	<10
Limit of detection (ng L^−1^)	0.137	14	0.38	1.8
Sample volume (mL)	8	5	200	1000
Extraction time	5 min	17 min	5 min	24 h
SPE configuration	Sampling stick	Online microcolumn	Dispersive microparticles	Dispersive microparticles
Reagent-free procedure	Yes	No	No	No

## Supplementary Materials

The following are available online at https://www.mdpi.com/2079-4991/11/2/512/s1: Figure S1: Accumulation of mercury traces onto new AuNP@TiO_2_ dipstick (a) in absence and (b–d) in presence of different concentrations of DOC (b) 5 mg L^−1^, (c) 10 mg L^−1^, (d) 15 mg L^−1^ DOC), Figure S2: Accumulation of mercury traces onto AuNP@SiO_2_ dipstick (a) in absence and (b–d) in presence of different concentrations of DOC (b) 5 mg L^−1^, (c) 10 mg L^−1^, (d) 15 mg L^−1^ DOC).
