# Supplementary material for: Photoactive Titanium Dioxide Films with Embedded Gold Nanoparticles for Quantitative Determination of Mercury Traces in Humic Matter-Containing Freshwaters
Source: Nanomaterials (Basel). 2021 Feb 18;11(2):512. doi: 10.3390/nano11020512 (PMC7921970; doi:10.3390/nano11020512)
Supplement: Supplementary file 1 [file nanomaterials-11-00512-s001.pdf]

nanomaterials

MDPI

Supplementary Materials

Photoactive Titanium Dioxide Films with Embedded Gold Nanoparticles for Quantitative Determination of Mercury Traces in Humic Matter-Containing Freshwaters

Vivian Stock [1,†], Anna Mutschler [2,†], Mika Lindén [2,*] and Kerstin Leopold [1,*]

[1] Department of Analytical and Bioanalytical Chemistry, Ulm University, Albert-Einstein-Allee 11, 89081 Ulm, Germany; vivian.stock@uni-ulm.de
[2] Department of Inorganic Chemistry II, Ulm University, Albert-Einstein-Allee 11, 89081 Ulm, Germany; anna.mutschler@t-online.de
* Correspondence: mika.linden@uni-ulm.de (M.L.); kerstin.leopold@uni-ulm.de (K.L.)
† Those authors contributed equally to this work.

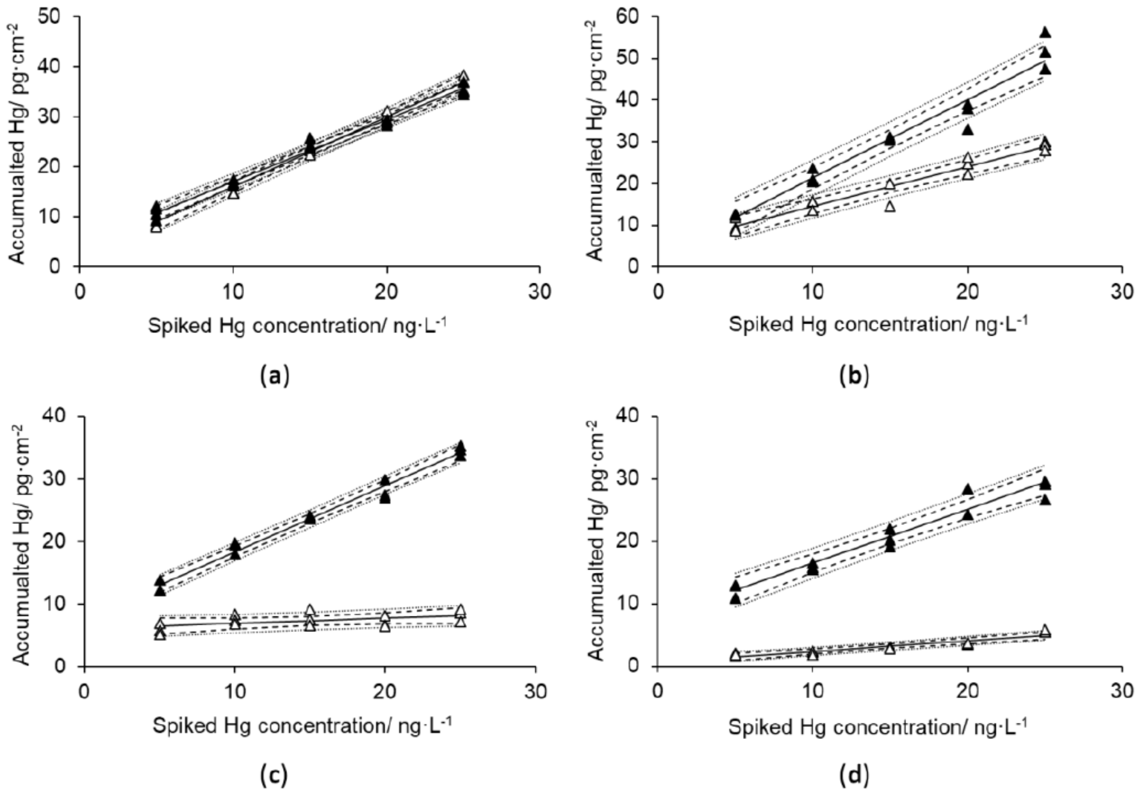

Figure S1. Accumulation of mercury traces onto new AuNP@TiO_2 dipstick (**a**) in absence and (**b–d**) in presence of different concentrations of DOC (**b**) 5 mg L^{-1}, (**c**) 10 mg L^{-1}, (**d**) 15 mg L^{-1} DOC. Filled symbols represent experiments with UV irradiation and empty symbols without UV irradiation during accumulation. Solid lines represent linear regression, dashed lines represent confidence interval, and dotted grey lines represent prediction intervals with $P = 95\%$ and $n = 15$. (Sample volume: 8 mL; Accumulation time: 5 min; Shaking speed: 230 rpm; Active dipstick area: 1.53 cm^2.).

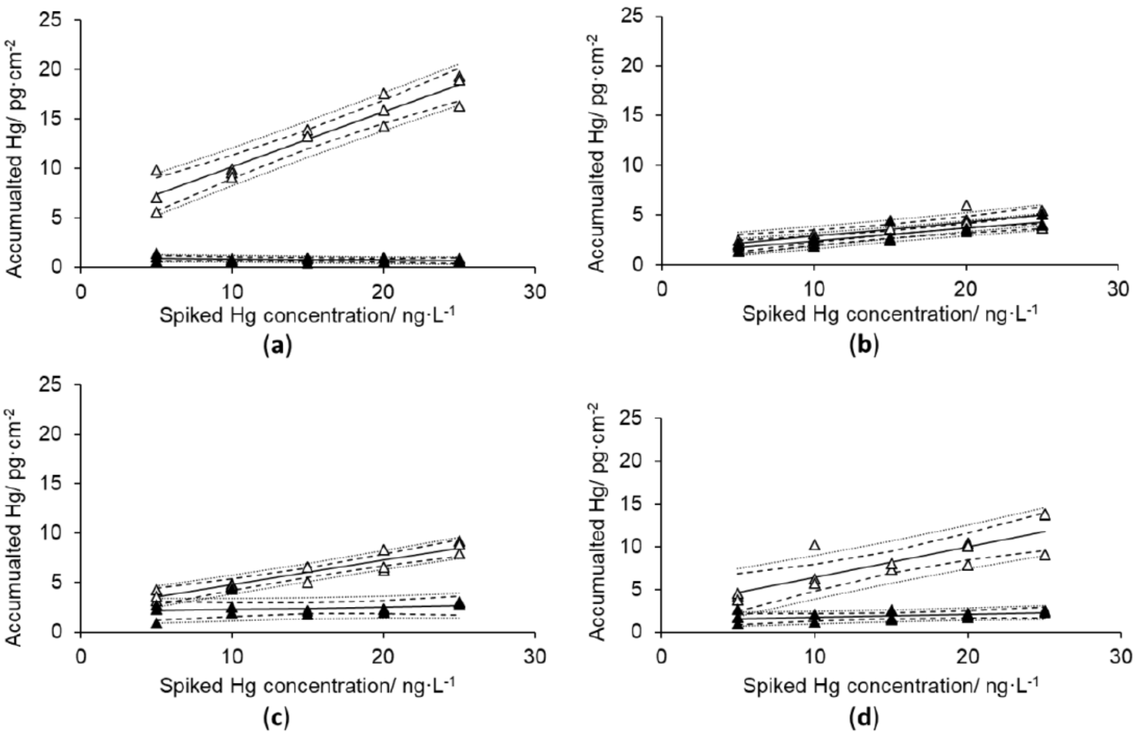

Figure S2. Accumulation of mercury traces onto AuNP@SiO_2 dipstick (**a**) in absence and (**b–d**) in presence of different concentrations of DOC (**b**) 5 mg L^{-1}, (**c**) 10 mg L^{-1}, (**d**) 15 mg L^{-1} DOC. Filled symbols represent experiments with UV irradiation and empty symbols without UV irradiation during accumulation. Solid lines represent linear regression, dashed lines represent confidence interval, and dotted grey lines represent prediction intervals with $P = 95\%$ and $n = 15$. (Sample volume: 8 mL; Accumulation time: 5 min; Shaking speed: 230 rpm; Active dipstick area: 1.53 cm^2.).

© 2021 by the authors. Licensee MDPI, Basel, Switzerland. This article is an open access article distributed under the terms and conditions of the Creative Commons Attribution (CC BY) license (http://creativecommons.org/licenses/by/4.0/).
